# The scientific basis of synergy in traditional Chinese medicine: physicochemical, pharmacokinetic, and pharmacodynamic perspectives

**DOI:** 10.1186/s13020-025-01291-y

**Published:** 2026-01-08

**Authors:** Xi Wang, Xiaoying Shi, Zhexing Xi, Zhitong Zhang, Zichen Luo, Jin Wang, Jinjun Shan

**Affiliations:** 1 Jiangsu Key Laboratory of Children’s Health and Chinese Medicine, State Key Laboratory On Technologies for Chinese Medicine Pharmaceutical Process Control and Intelligent Manufacture, Nanjing University of Chinese Medicine, Nanjing, 210023 China; 2 Medical Metabolomics Center, Nanjing University of Chinese Medicine, Nanjing, 210023 China; 3 College of Literature in Chinese Medicine, Nanjing University of Chinese Medicine, Nanjing, 210023 China

**Keywords:** Traditional Chinese medicine, Synergy, Target, Physicochemistry, Pharmacokinetics, Pharmacodynamics, Phase state, Supramolecular assembly

## Abstract

Traditional Chinese medicine, with millennia of history, demonstrates significant therapeutic efficacy against diverse diseases. A key characteristic of traditional Chinese medicine lies in the use of compound formulas—multi-herb concoctions that enhance efficacy and mitigate toxicity. The synergy within these formulas arises fundamentally from interactions among multiple active components. Recently, growing experimental studies have aimed to elucidate the scientific principles underlying synergy in traditional Chinese medicine. By reviewing literature over the past 30 years, this review summarizes that traditional Chinese medicine synergy manifests primarily through three key mechanisms: physicochemical interactions, pharmacokinetic processes, and pharmacodynamic effects. Furthermore, it provides an overview of methodological advances for studying these mechanisms, including HPLC fingerprinting, network pharmacology, and metabolomics, among others. Finally, it highlights current research limitations as well as challenges in traditional Chinese medicine modernization. This review systematically synthesizes current knowledge on traditional Chinese medicine synergy to establish a foundation for compatibility research and promote evidence-based clinical application.

## Introduction

As empirical medicine with a long history, Traditional Chinese Medicine (TCM) has demonstrated remarkable treatment efficacy in a wide range of diseases [1, 2]. Its value and potential have gradually gained global recognition. In clinical practice, TCM practitioners usually prescribe compound formulas consisting of two or more herbs, rather than single herbs, to achieve enhanced therapeutic effects [3]. These are a distinctive feature and fundamental therapeutic approach within TCM. Over thousands of years, numerous herbal combinations have been developed. These compound formulas improve disease prevention and treatment, broaden therapeutic ranges, and increase medication safety [4–6].

Synergy refers to a phenomenon wherein the combined use of two or more TCMs not only produces a therapeutic effect significantly exceeding the arithmetic sum of their individual effects but also alleviates the toxicity associated with each individual medicine when administered alone [7]. This fundamentally contrasts with additive effects, where the total effect equals the sum of individual contributions without mutual pharmacological interaction. The synergistic effects of TCM compound formulas are microscopically reflected in the alleviation of disease symptoms. This improvement arises from the active components targeting specific sites within the human body at a microscopic level [8]. The active components of various herbs in TCM compound formulas can interact extensively, potentially enhancing the final therapeutic effects [9]. Therefore, the combinatorial effects of multiple components represent the essence of TCM synergy.

TCM exerts its pharmacological effects at the microscopic level through the actions of bioactive components on the organism; thus, the essence of synergism lies in these components’ coordinated action. Notably, in TCM compound formulas, interactions may exist between components of different herbs or within a single herb. However, this study focuses primarily on compound-level synergistic and toxicity-attenuating mechanisms among different herbs. As TCM clinical practice predominantly uses compound formulas, this research helps elucidate TCM’s scientific basis.

Current research has achieved a profound understanding of the active constituents, specific therapeutic targets, and pharmacological activities within individual TCM herbs, such as extracting ginsenoside Rc from *Ginseng Radix et Rhizoma* [10] and astragalus polysaccharide from *Astragali Radix* [11]. However, existing studies have yet to fully unravel the complex mechanisms of multi-herb synergy, and a comprehensive, systems-level understanding is still lacking. These theories are vital for guiding the combination and usage of these herbs. Therefore, it is crucial to elucidate the combined effects of multiple components in TCM compound formulas through technologies such as network pharmacology [12], metabolomics [8], and so on. These approaches can reveal the scientific connotations underlying TCM theories and serve as a bridge connecting TCM with modern medicine. The combinatorial effects of active components in TCM can be interpreted from three perspectives: physicochemistry, pharmacokinetics, and pharmacodynamics.

Prior to entering the human body, interactions among active components can occur within the system of decoctions. These interactions occur through reactions such as supramolecular assembly and self-precipitation, leading to synergistic effects at the physicochemical level [13]. Subsequently, interactions during the in vivo transport and metabolism of active components can result in pharmacokinetic synergy [14, 15]. Target synergy refers to the synergistic effects that occur during the efficacious components of TCM reaching their sites of action and interacting with their respective targets [16]. Binding to the pharmacodynamic target initiates a cascade of downstream events that manifest as the observed pharmacodynamic effects. Lastly, exploring the theoretical basis of TCM compatibility from these three levels is very important for understanding and clarifying TCM’s essence to some extent.

**Fig. 1:**
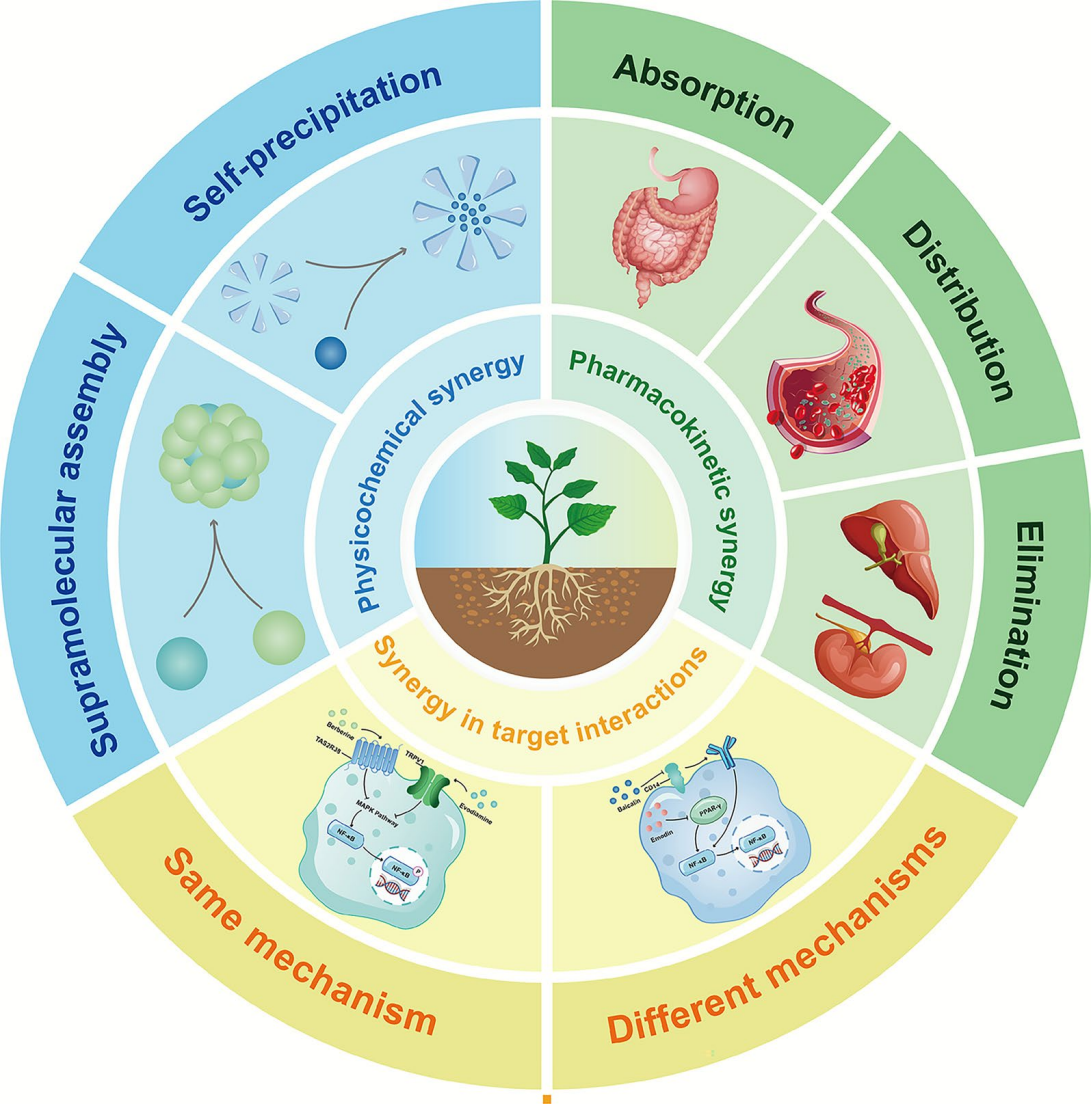
Summary diagram of the expression forms in TCM synergy. Physicochemical: altered dispersal triggers supramolecular assembly or self-precipitation. Pharmacokinetic: interactions during absorption, distribution, metabolism, and excretion interactions raise bioavailability. Pharmacodynamic: the same mechanism or different mechanisms

Significant progress has been made in this area, and a systematic review is necessary to summarize the accumulated knowledge. This article reviews the manifestations of synergistic effects in TCM from three different perspectives. It highlights the traditional use and methodological innovations that establish the foundation for research on synergy in TCM (Fig. 1).

## Method

Systematic literature search (1995–25 September 2025) was performed in PubMed, Web of Science Core Collection, ScienceDirect, CNKI, and Google Scholar without language restriction. The combined MeSH/free-text string was (“traditional Chinese medicine” OR “Chinese herbal formula” OR “herb pair”) AND (“synergy” OR “synergistic effect” OR “interaction”) AND (“pharmacokinetics” OR “pharmacodynamics” OR “physicochemical properties” OR “decoction”). Reference lists and conference abstracts were hand-searched for additional reports.

After duplicate removal, two reviewers independently screened titles/abstracts and then full texts; conflicts were resolved by a third reviewer. Studies were included if they were original in vitro, in vivo, or ex vivo investigations that explicitly tested synergy within multi-herb TCM preparations. Single-herb studies, reviews, patents, editorials, and multi-herb studies lacking synergy analysis were excluded.

Findings were synthesized narratively with study design, TCM composition, dosage form, analytical indicators, and key pharmacological/physicochemical outcomes.

## Manifestations of synergistic effects in TCM

### Physicochemical synergy

TCM compound formulas comprise complex constituents, including organic small molecules (e.g., flavonoids, terpenes, steroids, polyphenols), macromolecules (e.g., polysaccharides and proteins), and inorganic elements [17]. The efficacy of these active ingredients depends critically on their phase state, which governs their therapeutic impact.

Alterations in the initial dispersion state induce synergistic physicochemical effects that enhance curative performance, primarily manifested through supramolecular assembly and self-precipitation. Interestingly, these two forms can coexist within herbal pair compatibility, contributing to enhanced efficacy and attenuated toxicity.

Moreover, the unique decoction process of TCM promotes these components to form physicochemical polymorphisms such as real solutions, colloids, emulsions, or suspensions [17]. Wan et al. [18] found that the impurities removed by centrifugation from decoctions were mainly nanoscale particles, which could lead to the loss of water-soluble and lipid-soluble components. Moreover, the alcohol precipitation process results in the loss of active components as they precipitate out with a large amount of sediment [19]. Therefore, it is essential to evaluate whether processes such as alcohol precipitation and filtration compromise the physicochemical synergies of TCM. By disrupting the inherent phase states of decoctions, potentially diminishing the overall efficacy of compound formulas.

Unfortunately, many studies primarily rely on in vitro models, lacking robust clinical support. Furthermore, the biological activity assessments often use simplified cell-based assays, which may not fully capture the complex pathophysiology of diseases or the holistic efficacy of TCM. Therefore, future research should employ more physiologically relevant in vivo disease models and broaden their scope to TCM-specific decoction methods and more herbal pairs to confirm the pharmacological significance of self-precipitates.

### Supramolecular assembly

**Fig. 2:**
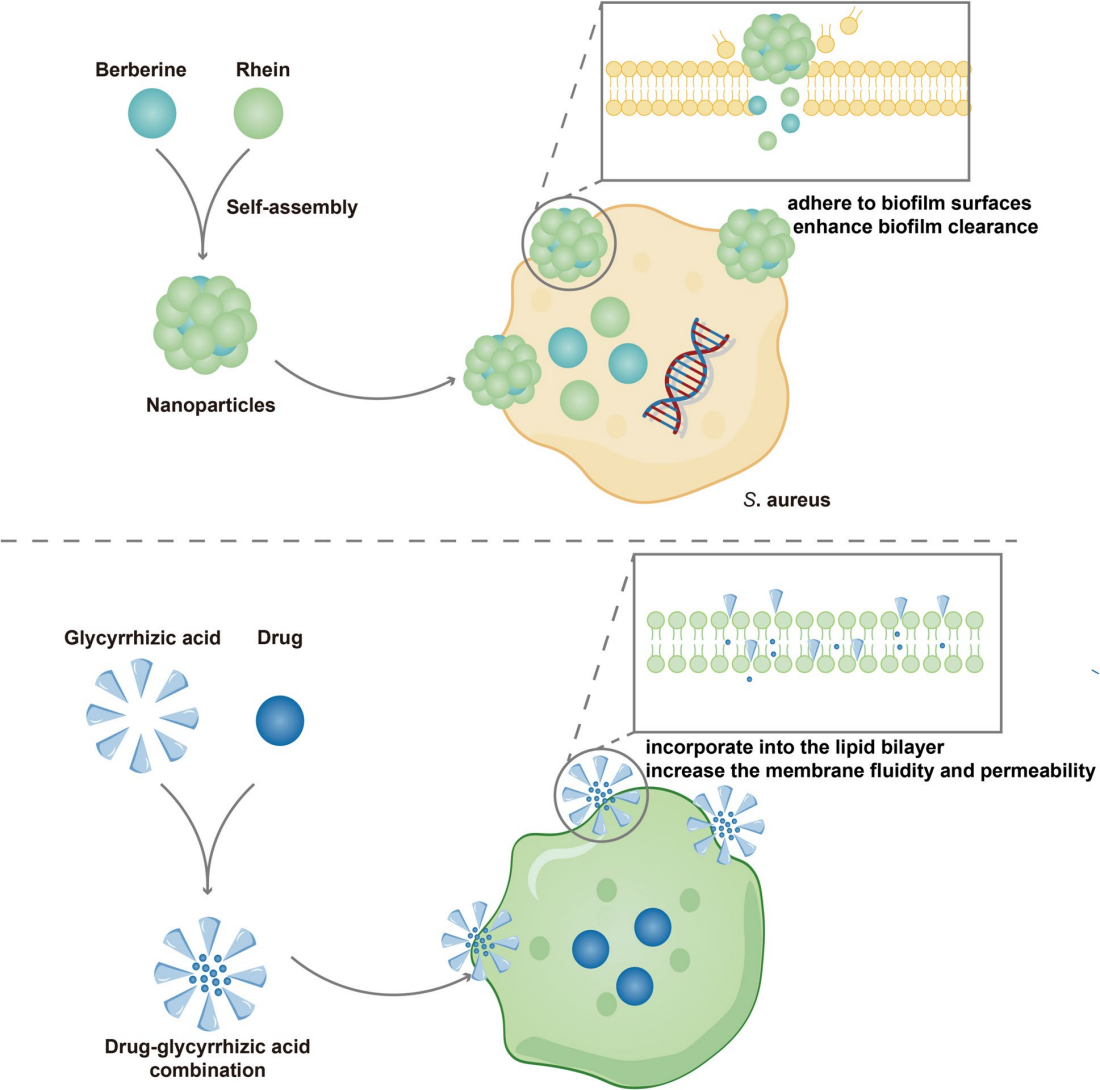
Two forms of supramolecular assembly in Chinese medicine compatibility. Electrostatics align Scutellaria glycosides with berberine into 1-D chains that collapse via hydrophobics into 3-D nanostructures; the particles bind and disrupt Staphylococcus aureus biofilms. Glycyrrhizic acid cages the actives in host–guest complexes that insert into lipid bilayers, increasing membrane fluidity and permeability

During the decoction of TCM, small molecules and large molecular components interact through non-covalent bonds, including hydrogen bonding and hydrophobic interactions. This results in the formation of supramolecular assemblies [20], affecting the phase states of the components (Fig. 2). Glycyrrhizic acid encapsulates hydrophobic molecules within its cyclic plane, creating host–guest inclusion compounds. The compounds can integrate into lipid bilayers, increasing membrane fluidity and permeability [21]. Furthermore, some macromolecules with low bioactivity, such as amylose in Gegen Qinlian decoction, undergo gelatinization during high-temperature decoction. This allows them to bind strongly to berberine from *Coptidis Rhizoma*, thereby improving its solubility [22].

Moreover, numerous studies have demonstrated that these supramolecular assemblies act as natural carriers that enhance ingredient dissolution, absorption, and efficacy, while also reducing potential toxicity [20, 23]. A clear example is the interaction between flavonoid glycosides from *Scutellaria Radix* and berberine from *Coptidis Rhizoma*. They first form one-dimensional complexes through electrostatic attraction. These then self-assemble into three-dimensional structures driven by hydrophobic interactions. These structures show stronger adhesion to Staphylococcus aureus biofilms, which improves biofilm clearance and antibacterial effects [23]. Another common herbal pair, *Coptidis Rhizoma* and *Rhei Radix et Rhizoma*, also demonstrates interesting assembly behavior. Tian et al. [24] found that rhein forms a layered structure in solution due to hydrogen bonding and self-assembles into nanoparticles with berberine through π–π stacking and electrostatic interactions, forming stable three-dimensional layered sediments in the aqueous phase [24]. Moreover, these nanoparticles, compared to the individual components, can adhere to the surface of the pathogenic bacterium Staphylococcus aureus, enhancing the clearance of the cell membrane by increasing the local concentration of berberine and rhein [24]. Further research has shown that this combined decoction reduces bitter taste and colon irritation compared to single herb extracts [25]. This may be due to the supramolecular structure slowing down the release of berberine [25]. Aristolochic acid is one of the components in traditional herbs such as *Akebia Caulis* and *Aristolochia*, but this component has side effects like nephrotoxicity, mutagenicity, and carcinogenicity [26]. However, the hydroxyl group of aristolochic acid and the quaternary ammonium ion of berberine, an active component in *Coptidis Rhizomas*, generate an electrostatic attraction, and through electrostatic attraction and π-π stacking, they self-assemble into a supramolecular structure, blocking the carboxyl site of aristolochic acid from being metabolized into the toxic aristolactam, providing a new perspective on the scientific rationale for the detoxification effect of TCM compatibility [27]. Similar self-assembly effects have been observed between *Coptidis Rhizoma* and *Cinnamomi Cortex* [28].

### Self-precipitation

During the decoction of certain, new precipitates are formed, which are generally composed of complex ingredients and may possess biological activity [29]. The Huanglian Jiedu Decoction, a renowned compound formula in TCM, encounters pronounced self-precipitation during the boiling process, primarily due to the interaction between *Coptidis Rhizoma* and *Scutellaria Radix*, with a self-precipitation solubility rate of 2.63% ± 0.20% [30]. In the decoction, electron transfer occurs between the carboxyl protons on acidic components, such as baicalin and the lone pair of electrons on the nitrogen atoms of basic components like berberine, leading to the formation of self-precipitates [31]. Research has shown that the self-precipitates from *Scutellaria Radix* and *Coptidis Rhizomas* significantly mitigate morphological damage and suppress apoptosis in nerve cells more effectively than the supernatant [32], indicating that these two herbs may synergistically enhance neuroprotective effects through self-precipitation.

Similarly, Aconiti Lateralis Radix Praeparata contains toxic aconite alkaloids that limit its clinical use [32]. Zhang et al. [33] analyzed the precipitates in the co-decoction of *Aconiti Lateralis Radix Praeparata* and *Glycyrrhizae Radix et Rhizoma* contain substantial aconitine, as detected by LC–MS. It is proposed that glycyrrhizic acid interacts with aconitine via acid–base reaction, forming precipitates that reduce toxicity [34].

Nevertheless, studies on the chemical composition of the self-precipitates in TCM compound formulas have revealed that they are consistent with the components found in the supernatant. This may result from non-covalent bonds within precipitates that are disrupted during chromatographic separation [29]. Therefore, this area warrants further investigation in the future.

### Pharmacokinetic synergy

Interactions among herbal constituents can alter the distribution and dynamics of active compounds, thereby enhancing therapeutic outcomes—an effect known as pharmacokinetic synergy in TCM. Due to the diversity of TCM components, the synergistic effects can be manifested in various aspects.

Medicament concentration typically increases, reaches equilibrium, and eventually declines. Insufficient concentration may result in no therapeutic effect, but conversely, excessive levels can lead to toxicity [35]. Thus, pharmacokinetic processes profoundly influence both efficacy and adverse effects by regulating ingredient distribution and concentration over time [36]. It is important to note that in pharmacokinetic studies of TCM formulations, increased systemic exposure—such as elevated AUC (area under the curve) or Cmax (maximum plasma concentration)—does not invariably confer therapeutic benefits. Instead, it may push plasma concentrations beyond the therapeutic window, increasing the risk of dose-dependent toxicity [37].

**Fig. 3:**
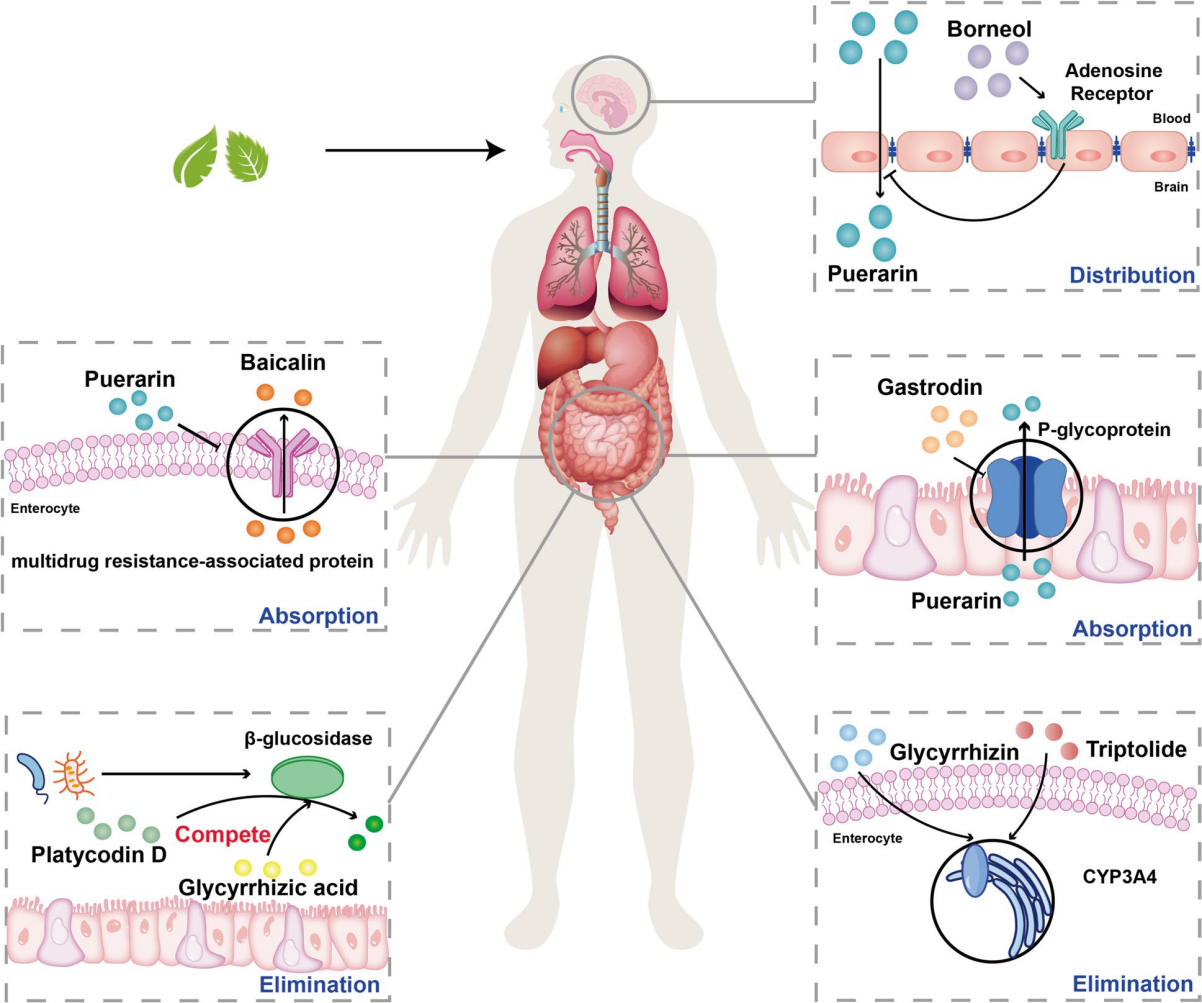
Mechanisms of pharmacokinetic synergy in representative herbal pairs through absorption, distribution, and elimination processes. Absorption: Gastrodin boosts puerarin solubility/inhibits P-gp reduces efflux and puerarin inhibits multidrug resistance protein 2 (MRP2) to increase baicalin retention/bioavailability. Distribution: Borneol activates adenosine receptors to promote puerarin brain delivery. Elimination: Glycyrrhizic acid competes for β-glucosidase to protect platycodin D from deglycosylation. Glycyrrhizin induces CYP3A to accelerate triptolide methylesterol metabolism/detoxification

Dr. As complex therapeutic systems, TCM (Traditional Chinese Medicine) compound formulations undergo analogous pharmacokinetic processes [38–40] (Fig. 3). Interactions among herbal constituents can alter the distribution and dynamics of active compounds, thereby enhancing therapeutic outcomes—an effect known as pharmacokinetic synergy in TCM. Due to the diversity of TCM components, the synergistic effects can be manifested in various aspects [41]. For example, the combination of Glycyrrhizae Radix et Rhizoma and Platycodonis Radix not only improves the bioavailability of compounds such as liquiritin and glycyrrhizic acid but also inhibits the metabolism of Glycyrrhizae Radix et Rhizoma’s active components via saponins from Platycodonis Radix [42], thereby potentially improving therapeutic outcomes. Another example is the Danshen–Honghua combination: it exerts a synergistic effect in activating blood circulation and resolving stasis. Their co-administration enhances the distribution and absorption of active components, reduces their clearance rate, and prolongs the half-lives of protocatechualdehyde and caffeic acid [43].

Therefore, a pharmacokinetic perspective offers valuable insights into the pharmacological behavior and interactive mechanisms of TCM synergy. A significant limitation lies in extrapolating PK parameters from animal studies directly to human therapeutic outcomes. Moreover, many studies tend to report positive synergistic interactions while overlooking instances where PK interactions are antagonistic, and deeper interactions involving protein binding, tissue distribution, and enterobacterial metabolism are often neglected. Thus, more comprehensive PK-PD modeling studies that correlate multi-component PK changes with in vivo efficacy endpoints are needed.

### Synergy in absorption

Oral administration is a common route for TCM, and the gastrointestinal tract is the primary site to absorb TCM’s active components [44]. The absorption rate in the digestive tract is influenced by multiple factors, including compound solubility, transmembrane transport efficiency, and intestinal content [45, 46]. Key determinants of transmembrane permeability include molecular weight, oil–water partition coefficient, and solubility. Generally, herb components with small molecular weight and high lipophilicity are more likely to enter cells through simple diffusion, while small molecule components in TCM can change the permeability of the membrane. For instance, glycyrrhizin from *Glycyrrhizae Radix et Rhizoma* incorporates into lipid bilayers and enhances membrane fluidity, thereby promoting the absorption of other compounds [21]. Moreover, transport proteins play crucial roles in both facilitated diffusion and active transport. Transport proteins such as P-glycoprotein (P-gp) and multidrug resistance-associated proteins (MRPs) [47] are highly expressed in intestinal epithelial cells and can pump substances from inside the cell to the outside. These proteins are important factors affecting the absorption of TCM’s active components. Inhibition of these protein functions leads to an increase in the bioavailability of substrate drugs and vice versa [48]. Studies have found that many TCM components can adjust these transport proteins, such as most TCM flavonoids, which can adjust P-gp [49]. A common compatibility strategy in TCM is to combine herbs containing P-gp or MRP2 substrates with those that inhibit these efflux pumps, thereby increasing the absorption and bioavailability of active components. *Puerariae Lobatae Radix* and *Gastrodiae Rhizoma* are traditionally used together for cardiovascular and cerebrovascular diseases. Puerarin (from Pueraria) is primarily absorbed via passive diffusion at low concentrations, while gastrodin enhances its solubility and inhibits P-gp and MRP2, reducing puerarin efflux and improving its intestinal absorption [50, 51]. Baicalin is the main active component of *Scutellaria Radix*, but due to its low lipophilicity, it is poorly absorbed in the intestines [52]. When combined with *Puerariae Lobatae Radix*, puerarin inhibits MRP2-mediated efflux of baicalin, thereby increasing its bioavailability [53]. Additionally, Synergistic effects via absorption modulation have also been reported in other herb pairs, such as *Aconiti Radix* with *Glycyrrhizae Radix et Rhizoma* [54], as well as *Panax Ginseng* and *Aconite* [55], contributing to enhanced efficacy and reduced toxicity.

### Synergy in distribution

TCM must be absorbed into the bloodstream and reach the site of action to exert its effect. The synergism of TCM in the distribution process is mainly divided into two aspects. First, there are barriers such as “blood–brain”, “blood-eye”, and “blood-fetus” in the human body, which make it difficult for some efficacious ingredients to reach the site of action [56–58]. However, many TCM compounds can enhance penetration across the blood–brain barrier. For example, *Borneolum Syntheticum* can inhibit the expression of P-gp and release tight junction proteins. Active constituents of Moschus affect tight junction integrity, promoting drug transport across the blood–brain barrier [59]. Thus, combining such TCM herbs improves medicament distribution to these regions. Puerarin improves cerebrovascular function and ameliorates ischemic reperfusion injury [60]. Tetramethylpyrazine is one of the main active ingredients extracted from the *Chuanxiong Rhizoma*, which is effective in the treatment of central nervous system diseases [61]. However, their poor blood–brain barrier permeability results in low cerebral bioavailability, limiting clinical applications. When *Borneolum Syntheticum* is combined with the two ingredients, it can effectively activate the adenosine receptor and decrease the expression of the tight junction protein ZO-1. This activation increases the intracerebral distribution of puerarin and tetramethylpyrazine and facilitates the ingredients to take effect [62].

The combination of *Scutellaria Radix* and *Bupleuri Radix* targets the liver and lungs, and is commonly used to treat respiratory and digestive disorders. Compared to *Scutellaria Radix* alone, this combination significantly increases the concentrations of saponins from *Vinegar Bupleuri Radix* and baicalin from *Scutellaria Radix* in the liver, lungs, and spleen [63]. *Achyranthes Bidentata Radix* combined with *Phellodendri Chinensis Cortex* enhances the treatment of gouty arthritis by elevating joint concentrations of berberine [64]. The combination of *Gastrodiae Rhizoma* and *Chuanxiong Rhizoma* exhibited a synergistic effect, indicative of a potential for enhanced therapeutic outcomes. This combination demonstrates synergistic effects in plasma, promoting the accumulation of active compounds in brain tissue and increasing bioavailability [65].

### Synergy in elimination

Before entering systemic circulation, Chinese medicines undergo various degradation and metabolic processes. These include degradation by digestive secretions, biotransformation by gut microbiota, enzymatic processing in the gastrointestinal wall, and hepatic metabolism. These processes may generate new bioactive or toxic compounds. For example, ginsenoside Compound K, the major gastrointestinal metabolite of *Ginseng Radix et Rhizoma*, exhibits high membrane permeability, antitumor, and anti-inflammatory activities [66]. Synergistic effects during elimination primarily involve interactions with intestinal microbiota before systemic absorption [67]. Human intestinal microbiota abundantly produce β-glucosidase, which deglycosylates herbal glycosides, often reducing their bioavailability. In the *Glycyrrhizae Radix et Rhizoma* and *Platycodonis Radix* pair, glycyrrhizic acid competes with platycodin D for substrates that are deglycosylated in the gut, protecting platycodin D from decomposition, thereby increasing the bioavailability of the latter [14].

Herbal combinations can also modulate in vivo metabolism by influencing intestinal microbiota and gut morphology. For example, the combination of *Scutellaria Radix* and *Rhizoma Coptidis* treats ulcerative colitis (UC) more effectively than either herb alone by modulating intestinal flora diversity and increasing beneficial bacteria abundance [68]. In addition, the use of *Scutellaria Radix* in combination with *Sophorae Flos* may also synergize the treatment of hypertensive DN by improving the balance of the intestinal microbiota [69]. *Rhizoma Coptidis* can cause adverse clinical reactions, primarily due to the toxicity of its active constituent berberine [70]. Wang et al. found that combining *Rhizoma Coptidis* with *Vladimiriae Radix* alleviated mouse body weight loss, impaired intestinal motility, and reduced ileal crypt numbers induced by *Rhizoma Coptidis* alone. This combination, in contrast to using *Rhizoma Coptidis* alone, may counteract these effects by elevating serum adenosine and inosine [71].

Synergy also occurs in enterohepatic metabolism. CYP3A [72], which is highly expressed in the liver and small intestine, plays a key oxidative role in metabolizing herbal compounds. Herbal compatibility can modulate CYP3A activity, thereby enhancing efficacy and reducing toxicity. The Chinese herbs *Citri Reticulatae Pericarpium* and *Anemarrhenae Rhizoma* can be used to treat cough. Zhang et al. [73] reported that anemarsaponin BI from *Anemarrhenae Rhizoma* prolongs the half-life of nobiletin from *Citri Reticulatae Pericarpium* and enhances its metabolic stability via CYP3A inhibition, thereby improving therapeutic efficacy. Triptolide, the main effective substance of *Tripterygium Wilfordii*, is limited in its application due to its hepatotoxicity. Side effects such as cardiotoxicity and ototoxicity were observed [74]. Tai [75] found that when *Glycyrrhizae Radix Et Rhizoma* was combined with *Tripterygium Wilfordii*, Glycyrrhizin promotes the activity of CYP3A to accelerate the Triptolide methyl ester’s metabolic elimination from the body, which in turn reduces the toxicity.

The in vivo mechanisms of TCM encompass excretory processes, and the synergistic effects of these medicines are indicated through the analysis of bile, urine, feces, secretions, and exhaled gases [76]. These analyses help identify both the original herbal compounds and their biotransformation products in vivo.

## Synergy in pharmacodynamics

**Fig. 4:**
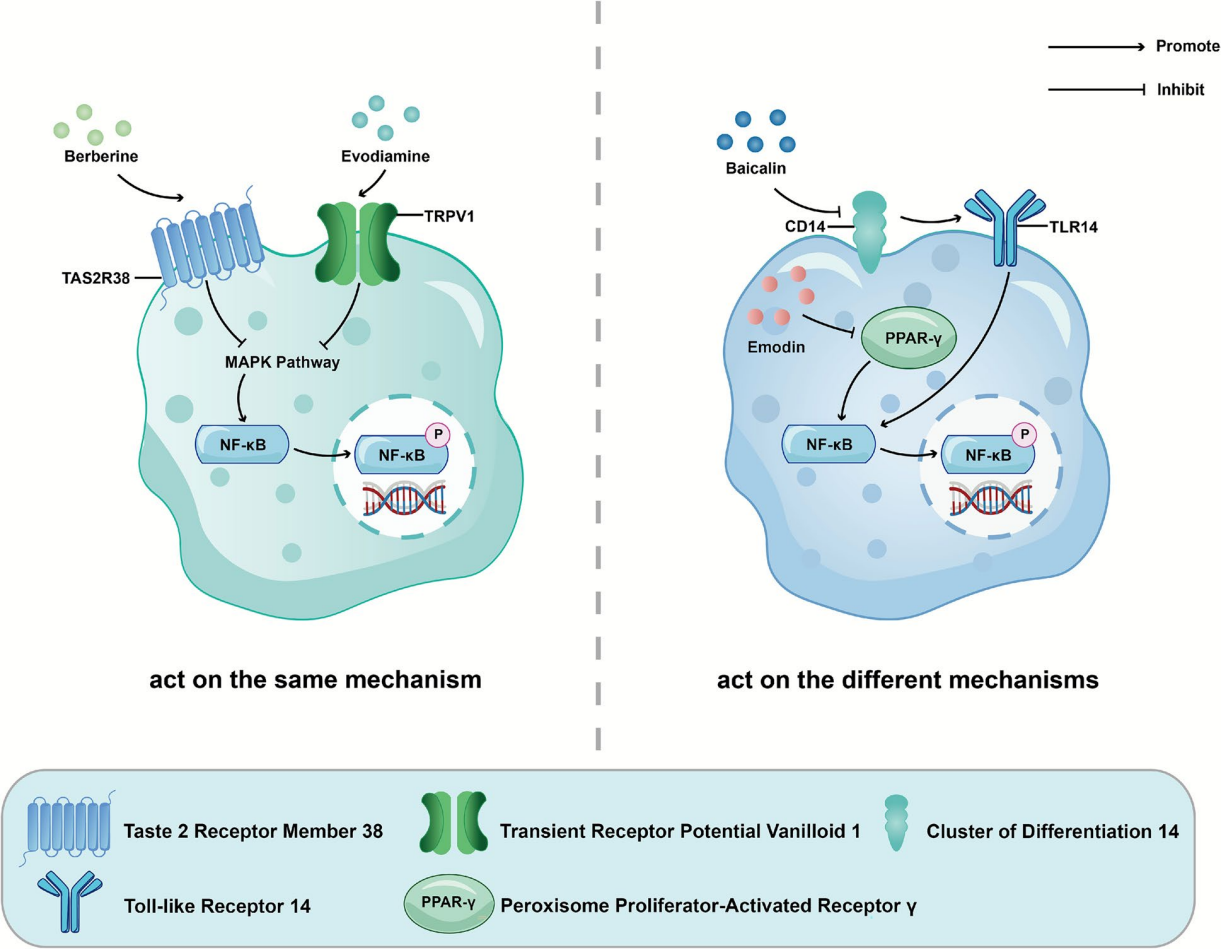
The synergy in pharmacodynamics. Components with the same mechanism or complementary mechanisms. *Euodiae Fructus*-*Coptidis Rhizoma*: Berberine and evodiamine both inhibit the MAPK pathway to suppress NF-κB phosphorylation and inflammation. *Rhei Radix et Rhizoma*-*Scutellariae Radix*: baicalin inhibits CD14 reduces TLR14 to suppress NF-κB/inflammation, while emodin inhibits PPAR-γ to synergistically suppress NF-κB phosphorylation

A well-recognized advantage of TCM compound formulas lies in their multi-targeted approach to treating diseases. Rather than a simple summation of individual constituents, the pharmacodynamic synergy arises from concerted interactions among bioactive substances that simultaneously engage distinct, high-affinity molecular targets. This synchronized occupancy initiates convergent or complementary signaling cascades, amplifying and fine-tuning downstream network responses that translate into the clinically observed, dose-sparing, and toxicity-reducing effects characteristic of TCM therapeutics [77, 78]. In 1996, Xue et al. created the “shotgun theory” as the earliest expression of multi-component and multi-target synergistic effects of TCM [79]. Extending this framework, Wu subsequently formulated the “disease-reduction effect” hypothesis, emphasizing that such synchronized target occupancy and convergent signaling cascades yield the synergy effect [80]. In 2014, Cai introduced the “effective form theory” within the context of TCM [81], suggesting that the collection or addition of the “effective forms” in TCM is the core material basis of pharmacodynamic effects. This theory elucidates that the “additive effect” of each pharmacodynamic substance concentration in the blood represents one of its mechanisms. Furthermore, it highlights the “toxicity scattering effect”. It is stated that the cumulative pharmacodynamic substances in the blood are identified as a key mechanism behind the efficacy of TCM. With the development of molecular biology, systems biology, and network pharmacology, we have clarified the association between therapeutic targets of diseases and the synergistic mechanisms of herbs. An increasing number of studies have revealed the synergistic effect of herb pairs on these mechanisms. Synergy in pharmacodynamics can be classified and interpreted based on whether the targets act through a unified mechanism (Fig. 4). Some herb pairs share the same mechanism to enhance the effect on this pathway. In contrast, other herb pairs exert multi-target effects through complementary mechanisms, which confer advantages in treating diseases with complex pathogenic mechanisms.

It should be noted that most cited studies have small sample sizes, limiting statistical power and result stability. After network pharmacology screening, no in vivo experimental verification was conducted—research only remained at the in vitro molecular docking stage, making it impossible to confirm whether “target binding” can truly translate into “in vivo effects” to exert synergy. Animal models also have limitations: their low matching degree with common clinical pathological types may affect the reliability of clinical translation of conclusions.

Future studies should optimize models, expand sample sizes, and improve the evidence chain to further enhance the rigor of research on TCM pharmacodynamic synergy and its clinical reference value.

### Synergism with the same mechanism

When the corresponding mechanism of a disease is clarified, two or more Chinese herbal medicines acting on the same pathway may potentiate the effect on this mechanism, thereby producing a synergistic effect. Neutrophil extracellular trap (NET) released by neutrophils is crucial for tumor metastasis [82]. *Rehmanniae Radix*-*Corni Fructus*, the classical herbal pair of Chinese compound formula, Liuwei Dihuang Pills, exert a significant protective effect on diabetic nephropathy (DN), with its synergistic mechanism being “target complementation and dual pathway blockage”. The primary active compounds—loganin (Log), Rg1, and catalpol (Cat)—either alone or in combination, effectively inhibited activation of the AGE-RAGE axis and its downstream signaling pathways, reducing lipid accumulation. Notably, Log attenuated p38 MAPK phosphorylation more significantly than Cat, while Cat markedly suppressed Nox4 expression. Furthermore, their combination inhibited the RAGE/p38 MAPK/p65 NF-κB and RAGE/Nox4/p65 NF-κB pathways, thereby blocking the AGE-RAGE axis and its downstream components (p38 MAPK, Nox4). This inhibition mitigated podocyte injury and apoptosis, alleviated proteinuria, and delayed the onset and progression of DN. Importantly, the combined treatment exerted a more significant inhibitory effect than single-agent treatment [83].

Berberine, the main active ingredient of *Coptidis Rhizoma*, and Evodiine (EVO), the main ingredient of *Euodiae Fructus*, are known to activate TAS2Rs and TRPV1 respectively and can be used in the treatment of gastroesophageal reflux disease (GERD). Cui et al. [84] found that berberine can activate TAS2Rs to reduce the phosphorylation of p65, JNK, and ERK through the MAPK/NF-κB signaling pathway, thereby exerting its anti-inflammatory and barrier-protective effects to reduce mucosal damage. EVO protects the esophageal mucosa by reducing inflammation and suppressing the MAPK/NF-κB signaling pathway through TRPV1. The synergism of the two active components inhibits the MAPK/NF-κB pathway in GERD rats and regulates macrophage polarization through TAS2Rs/TRPV1 targets. *Paeoniae Radix Rubra*-*Aconiti Lateralis Radix Praeparata* is widely used in TCM to treat liver disease. Studies have found that it can promote macrophage M2 polarization, inhibit M1 polarization, restore M1/M2 immune balance to reduce inflammation, restore immune balance, and prevent the intensification of liver failure response [85].

Yinxieling formula (YF) is a TCM compound that has a long history and has traditionally been used in the treatment of psoriasis due to its anti-inflammatory and immunomodulatory properties. Zeng et al. [86] found that YF extract significantly reduced the production of inflammatory factors induced by LPS in RAW264.7 cells, identified the extracts with key targets, and identified the active components such as umbelliferolactone, which was the main active component of *Atracylodis Macrocephalae Rhizoma*, and Vanillic acid, gentianic acid, and echinoside, which are the main components of *Paeoniae Radix Rubra*, *Mume Fructus*, and *Rehmanniae Radix* respectively. Previous studies have shown that these components mainly exhibit anti-inflammatory and antioxidant properties, and the authors conducted a combination and functional verification to confirm that the herbal pair can inhibit the production of LPS-induced IL-23, suggesting that the herbs in YF synergistically act on the anti-inflammatory mechanism.

In the treatment of ovarian cancer, the combination of *Scutellariae Barbatae Herba* and *Scleromitrion Diffusum* [87] can affect the expression of genes related to heme catabolism and ferritin autophagy in A2780 cells, and these two herbs can synergically promote heme catabolism and ferritin autophagy. Furthermore, several studies have accurately addressed the compatibility of TCM, demonstrating its synergistic enhancement effects. The herbal pair *Trichosanthis Fructus* and *Allii Macrostemonis Bulbus* is frequently utilized in practice. Bao et al. [88] discovered that this combination can down-regulate the expression of P2RY12, and confirmed through a dual luciferase reporter gene analysis system that the compounds within this pair significantly reduce P2RY12 levels. These findings suggest that the *Trichosanthis Fructus*-*Allii Macrostemonis Bulbus* herbal pair can synergistically regulate the lipid phagocytosis of VCMN-derived foam cells through the P2RY12 node, thereby preventing atherosclerosis.

In clinical practice, *Glycyrrhizae Radix et Rhizoma* and *Pinelliae Rhizoma* were frequently used to treat pneumonia. Luo et al. [89] used network pharmacological methods to initially explore the key hub nodes and active ingredients of this herbal pair. Molecular docking verification of IL-6 and STAT3, two of the most critical nodes in the protein–protein interaction network, showed that the *Glycyrrhizae Radix et Rhizoma* and *Pinelliae Rhizoma* pair can directly bind with both IL-6 and STAT3 nodes to reduce inflammation. *Stephania Tetrandra*-*Astragalus Membranaceus* is a classic herbal pair widely used in the treatment of nephrotic syndrome. Xu et al. [90] revealed the synergistic down-regulation of AQP2 and AVP gene expression through animal experiments and metabolomics. Regulating water channel protein can reduce edema, and can also relieve the inflammation of nephrotic syndrome rats by regulating the metabolism of methionine and cysteine, and the effect of combined use is greater than that of a single herb. The study found that miRNA-34a-5p can directly regulate the role of SMAD2 in osteoblast differentiation. At the same time, Chinese medicines *Curculiginis Rhizoma* and *Epimedh Folium* can restore the expression of miRNA-34a-5p and synergistically inhibit the expression of SMAD2, thereby alleviating LPS-induced bone loss [91].

### Synergism with different mechanisms

The compatibility of Chinese medicine can treat diseases synergistically by interacting with different mechanisms. Multi-target synergy is particularly suitable for diseases with relatively complex pathogenesis. The herbal pair composed of *Rhei radix et rhizoma* and *Coptis Rhizoma* has a synergistic effect in the treatment of colitis. Colitis has a complex pathogenesis, involving multiple factors such as intestinal flora imbalance and immune dysregulation [92]. Yan et al. [93] investigated the therapeutic mechanism of the herb pair. They found that *Rhei Radix et Rhizoma* promotes HIF-1α degradation, while *Coptis Rhizoma* inhibits the MAPK pathway; additionally, the two herbs bind to different domains of AKT1 in a non-competitive manner, enhancing the pathway inhibitory effect. Furthermore, Xu et al. [94] found that combined treatment of baicalin and emodin could reduce intestinal inflammation in mice with colitis induced by sodium dextran sulfate, and the effect was better than that of baicalin or emodin given alone. When combined, baicalein decreased the expression of CD14, while emodin increased the expression of PPAR-γ, thereby inhibiting the activity of p50 on the NF-κB pathway, resulting in a synergistic effect on anti-inflammation.

Both *Ginseng Radix et Rhizoma* and *Salviae Miltiorrhizae Radix et Rhizoma* have anti-tumor metastasis effects “dual-link blockage”. Cryptotanshinone inhibits neutrophil recruitment to metastatic sites by down-regulating CD62E expression. Ginsenoside Rg1 inhibits the formation of NETs, which are neutrophilic extracellular structures by inhibiting the production of ROS and the activation of ERK1/2 and MAPK signaling pathways, thus reversing the pro-tumor effect of NETs [95]. The effect of both is enhanced. Nephrotic syndrome exhibits an extremely complex pathogenesis, triggered by multiple etiologies and involving disorders of the immune-inflammatory network, metabolic imbalances, and other factors [96]. *Imperatae Rhizoma* and *Hedyotis diffusa Willd.* herbal pair achieves multi-targeted, multi-pathway intervention for therapeutic effects against nephrotic syndrome through the synergistic action of dual mechanisms: anti-inflammation (via the NF-κB pathway) and lipid-lowering (via the PPARγ-CYP7B1 and PCSK9-LDLR pathways) [97].

Huashi Baidu formula (HBF) is often used to treat DN in clinics. Huang et al. [98] verified HBF’s renal protective effect via animal experiments, confirming its mechanism involves Apelin/SphK1/NF-κB-mediated oxidative stress, immunoinflammatory response, and Apelin/PAI-1/TGF-β-mediated extracellular matrix (ECM) formation. Target identification showed six compounds from *Paeonia, Astragali Radix*, *Rhei Radix et Rhizoma*, and *Glycyrrhizae Radix et Rhizoma* target and inhibit SphK1; while glycyrrhizoflavone, licochalcone B, and isoliquiritin from *Glycyrrhizae Radix et Rhizoma* target and inhibit PAI-1, thus suppressing ECM production. This suggests that herbs in HBF protect renal function via multi-target and multi-pathway co-inhibition.

Matrine and glycyrrhizin are representative active components of clinically used TCM. Hepatocellular carcinoma is highly complex, with its pathogenesis driven by viral factors, metabolic abnormalities, and immune microenvironment dysregulation—most cases are linked to underlying chronic liver disease [99]. The core of the herb pair’s synergy lies in “complementation between anti-tumor and liver-protective mechanisms”. Matrine has an anti-tumor pharmacological effect, and glycyrrhizin has a liver protection effect. Studies have shown that matrine can inhibit the migration and proliferation of cancer cells. Glycyrrhizic acid can effectively inhibit hepatocyte apoptosis. Han et al. [100] analyzed the mechanism of marsinine and glycyrrhizic acid in the treatment of liver cancer through network pharmacology and found that the two had synergistic effects on molecular pathways such as the NF-κB signaling pathway, MAPK/ERK signaling pathway, and TNF signaling pathway. Serological test results also indicated that the effect of the combination of two herbs was significantly better than that of single herb administration.

Metabolic-associated steatotic liver disease is driven by multiple pathways, including lipid metabolism dysregulation, inflammation-fibrosis, and ferroptosis [101]. The combination of curcumin (Cur) and resveratrol (Res) can synergistically regulate metabolism and apoptosis. It has been proven that Res can affect lipid metabolism by regulating the AMPK/mTOR signaling pathway, while Cur can regulate the mechanism of apoptosis. He et al. [102] studied the combination of Cur and Res and found that the synergistic effect of the two components can not only regulate the signaling pathway related to lipid metabolisms, such as the cascade of PI3K/AKT/mTOR and HIF-1/VEGF but also reduce the production of inflammatory mediators and oxidative stress factors at the same time, suggesting that Cur and Res can achieve synergistic effects from the two pharmacological effects of regulating metabolism and affecting apoptosis.

## Clinical translation of TCM synergy

Currently, clinical studies on synergistic herb pairs remain limited. To clearly illustrate the core differences between different herbal combinations, Table 1 summarizes the formulas and origins of representative herb pairs, along with their TCM mechanisms and corresponding modern clinical applications. This table intuitively demonstrates the “traditional efficacy-application” matching relationship. To further elaborate on its clinical translation value and provide references for the key directions of future research.

**Table 1:** Source compounds of Chinese herb pairs and their traditional applications

Herb pair	First source formulas for pairs of medicines	Antiquarian source	Traditional efficacy of medicinal pairs	Clinical application	References
*Bupleuri Radix-Paeoniae Radix Alba*	Sini San	Treatise on Cold Damage Diseases	Soothing the liver and regulating qi	Antidepressant	[16]
*Chuanxiong Rhizoma-Borneolum*	Suxiao Jiuxin pills	Experiential Prescriptions	Activating blood and moving qi to relieve pain, activating blood and resolving stasis, and inducing resuscitation	Against atherosclerosis, protecting the ischemic myocardium, regulating blood lipids, and dilating arteries	[125]
*Rhei Radix et Rhizoma-Coptidis Rhizoma*	Dahuang Huanglian Xiexin Decoction	Treatise on Cold Damage Diseases	Clearing heat and purging fire, detoxifying, combination of them can enhance efficiency and reduce toxicity	Treating antibiotic-associated diarrhea. Regulating the gut microbiota to repair the intestinal barrier and treating ulcerative colitis UC	[131, 132]
*Rehmanniae Radix-Cornifructus*	Liuwei Dihuang Pills	Key to Therapeutics of Children’s Diseases	tonifying the liver and kidneys	Treating diabetic nephropathy	[83]
*Stephaniae Tetrandrae Radix-Astragali Radix*	Fangji Hungqi Decoction	Synopsis of Prescriptions of the Golden Chamber	Tonifying qi fortifies the spleen, inducing diuresis to alleviate edema	Treating nephrotic syndrome	[90]
*Aconiti Lateralis Radix Praeparata-Glycyrrhizae Radix et Rhizoma*	Sini Decoction	Treatise on Cold Damage Diseases	Enhancing efficiency and reducing toxicity of Aconitii Lateralis Radix Praepartata	Treating hepatitis, liver injury, and liver failure, and protecting liver cells by reducing the content of taurine-conjugated bile acids. Treating rheumatoid arthritis, relieving joint pain, Alcoholic fatty liver, and Antitumor	[133–136]
*Glycyrrhizae Radix et Rhizoma-Pinelliae Rhizoma*	Maxing Shigan Decoction	Treatise on Cold Damage Diseases	Soothing the medicinal properties, reducing the toxicity of pinelliae rhizome, relieving cough, and dispelling sputum	Antiviral, anti-inflammatory, and immunomodulatory, preventing and treating COVID-19	[89]
*Phellodendri Chinensis Cortex-Achyranthis Bidentatae Radix*	Sanmiao Pill	Orthodox Transmission of Medicine	Tonifying the liver and kidney, strengthening sinew, and clearing the dampness-heat	Treating rheumatoid arthritis	[137]
*Glycyrrhizae Radix et Rhizoma-Platycodonis Radix*	Jiegeng Decoction	Treatise on Cold Damage Diseases	Clearing heat and detoxifying, promoting lung function, and clearing the throat	Treating acute lung injury and preventing pulmonary fibrosis	[138, 139]
*Puerariae Lobatae Radix-Coptidis Rhizoma*	Gegen Qinlian Decoction	Treatise on Cold Damage Diseases	Clearing heat and stopping diarrhoea	Treating ulcerative colitis, diabetes mellitus, and hyperlipidemia	[140]
*Coptidis Rhizoma-Glycyrrhizae Radix et Rhizoma*	Gancao Xiexin Decoction, Gegen Qinlian Decoction, Banxia Xiexin Decoction	Treatise on Cold Damage Diseases	Tonifying qi, clearing heat, and resolving masses	Treating obesity and insulin resistance	[141]
*Coptidis Rhizoma-Cinnamomi Cortex*	Jiaotai Pills	Han’s Medical Communication	Coordinating the heart and kidney, clearing heat, and calming the mind	Improving insomnia and its adverse effects, having antidepressant effects, and being antidiabetic	[142, 143]
*Coptidis Rhizoma-Evodiae Fructus*	Zuojin Pill	Danxi’s Experiential Therapy	Clearing and reducing liver fire, circulating and down-regulating qi to stop vomiting	Treating gastrointestinal diseases such as UC	[144, 145]
*Astragali Radix-Angelicae Sinensis Radix*	Danggui Buxie Decoction	Treatise on Clarification of Perplexities About Internal and External Damage	Tonifying qi to generate blood	Treating middle cerebral artery occlusion/reperfusion, improving menopausal syndrome, and promoting wound healing	[146–149]
*Scutellariae Barbatae Herba-Spreading Hedyotis Herb*	Xiaoliu Yin	Royal Surgeon Complete Book	Clearing heat and detoxifying、 dispersing abscesses and nodules	Anti-tumor, treating bladder cancer and ovarian cancer	[150, 151]
*Scutellariae Radix-Bupleuri Radix*	Xiaochaihu Decoction, Dachahu Decoction	Treatise on Cold Damage Diseases	Releasing the flesh and allaying a fever	Treating upper respiratory tract infections and the common cold	[152, 153]
*Scutellariae Radix-Coptidis Rhizoma*	Huanglian Jiedu Decoction	Handbook of Prescriptions for Emergencies	Clearing heat and detoxifying	Treating colitis and type 2 diabetes	[154, 155]
*Trichosanthis Fructus-Allii Macrostemonis Bulbus*	Gualou Xiebai Banxia Decoction	Synopsis of Prescriptions of the Golden Chamber	Unblocking yang to dissipate binds and moving qi to dissipate nodules	Relieving atherosclerosis, treating cardiorenal syndrome	[156]
*Ginseng Radix et Rhizoma-Aconitii Lateralis Radix Praepartata*	Sini Jiaren Shen Decoction	Treatise on Cold Damage Diseases	Restoring yang to save it from collapse	Treating shock, chronic heart failure	[157, 158]
*Gastrodiae Rhizoma-Uncariae Ramulus Cum Uncis*	Tianma Gouteng Decoction	A New Meaning of the Treatment of Miscellaneous Internal Diseases in Chinese Medicine	Soothing the liver, submerging yang, and clearing heat	Treating Alzheimer’s disease	[159]

## Research methods for synergy

### Methods for studying physicochemical synergies

The study of physical and chemical synergy primarily focuses on pH, solubility, apparent oil–water partition coefficient, and so on to determine the fundamental properties of the active components [103], among which solubility can be measured using methods like the solubility equilibrium method or synthesis method. For the components of herbal pairs, HPLC fingerprinting [104] or techniques like UFLC-IT-TOF/MS, as used by Yang et al. [105], can be employed to identify and compare the main chemical components in single herbs and herbal pairs like *Aconiti Lateralis Radix Praeparat* and *Glycyrrhizae Radix et Rhizoma*. The apparent oil–water partition coefficient can be determined using the *n*-octanol–water system [106].

Lei et al. [107] discovered that different concentrations of glycyrrhizic acid in the *Rhizoma Coptidis* and *Glycyrrhizae Radix Et Rhizoma* herbal pair could alter the oil–water partition coefficient of berberine hydrochloride. To separate physical and chemical components, techniques like gradient centrifugation, dialysis, ultrafiltration, or field flow fractionation are utilized [108], aiming to minimize damage to the physical morphology of the components. Component characterization can be performed using a granulometer to determine size [109], and electron microscopy for analysis of size and morphology. Chromatographic and mass spectrometric techniques are employed to analyze the composition of active components in TCM compound formulas [110], while spectroscopic methods and isothermal titration calorimetry [111] are used to characterize and predict the potential mechanisms of supramolecular formation in the components. For example, Liang et al. [112] used a comprehensive approach involving electron microscopy, UV–vis spectroscopy, FTIR spectroscopy, UHPLC-HR-MS, and molecular dynamics simulations to validate that the herbal pair of *Astragali Radix* and *Angelicae Sinensis Radix* can form stable supramolecular structures.

### Methods for studying synergy in pharmacokinetics

The study of in vivo processes can explore the synergistic effects of TCM components by investigating changes in the spatiotemporal distribution of active components before and after compatibility. For temporal distribution, pharmacokinetic experiments can be conducted to establish “concentration–time” curves for active components [113, 114]; for spatial distribution, tissue distribution studies can be performed [115]. YARO PETER [116] conducted pharmacokinetic research and detected that the peak blood concentrations of ginkgolides A, B, and bilobalide reached 1.5 h. The study also detected higher concentrations of these compounds in liver and kidney tissues compared to lower concentrations in the brain.

### Synergy in absorption

Research on absorption process synergy can be completed through pharmacodynamic experiments using animal or cell models. For example, a human colon adenocarcinoma Caco-2 cell monolayer model [117, 118] can be established to measure transmembrane resistance and transmembrane markers. This model can be used to investigate the impact of TCM compatibility on the transmembrane transport of active components across the small intestinal epithelium. An MDCK-MDR1 cell model [115] can be used to compare the components’ abilities to overcome the efflux effect of P-gp before and after the compatibility. Although cell models are limited in fully replicating the physiological conditions of the gastrointestinal tract, they can be effectively complemented by a range of in vitro and in vivo methodologies, including the isolated everted intestine technique [119], Using Chamber system [120], intestinal loop method [121], and in situ intestinal perfusion technique [122].

### Synergy in distribution

In vitro verification can be conducted using cell simulation to research the distribution processes, while it can be achieved through methods such as tissue distribution and in vivo tracer imaging [123]. For example, a co-culture model of brain capillary endothelial cells and astrocytes can be used to study the impact of herbal pairs on crossing the blood–brain barrier [124]. Compared with single herb treatments of either *Chuanxiong Rhizoma* or *Borneolum Syntheticum*, the combination of these two herbs significantly enhanced the relaxation effect on the vascular rings (*P* < 0.01) [125]. The BeWo cell model can be used to simulate the “blood-fetus” barrier [123]. Zhang used nobiletin to treat the human placental villous cancer cell line (BeWo cell line) to demonstrate its protective effect on trophoblast cell apoptosis.

### Synergy in elimination

To study the synergistic metabolic processes of herbal medicines in the gut and liver, animal or cellular models such as the human colon cancer TC7 cell model [126] can be used for comparing the altered metabolizing ability of CYP3A on efficacious components before and after the compatibility [72, 127]. For example, by comparing the activity of CYP3A4 and medicament distribution under different treatment conditions, it was proved that serpentine B in Compound Bushen Yizhi Formula could inhibit the activity of CYP3A4 enzyme, which in turn improved the bioavailability of its main active ingredients [128]. In addition, animal intestinal S9, small intestinal and liver sections, and intestinal and liver microsomes can be prepared for the comprehensive study of phase I and II metabolic enzymes. Liver metabolism in the physiological state has been studied using isolated and in vivo liver perfusion [129], as well as artificial intestinal fluids and anaerobic cultures of fecal commensal bacteria to simulate the intestinal luminal metabolism of components [130].

### Methods for studying synergy in pharmacodynamics

TCM compound formulas activate multiple targets. The complexity of mechanisms presents a challenge in comprehending the molecular mechanisms underlying the synergistic effects of TCM. Nowadays, there are various ways to study the synergy in pharmacodynamics with the development of molecular biology, network pharmacology, and other technologies. The main research approach is to examine the differences in the efficacy of Chinese herbal medicines or efficacious ingredients before and after the compatibility of in vivo and in vitro models [94]. The potential action targets or efficacious ingredients of various Chinese medicinal herbs are further identified through systems biology methods (transcriptomics, proteomics, metabolomics, etc.), high-throughput screening methods (biochip, biochromatography, etc.), and web-based pharmacological target prediction. Finally, validation is carried out using molecular biology and other means [68, 69, 71, 94]. For example, Li et al. [16], utilized findings from network pharmacology and metabolomics to study the combination of *Bupleuri Radix* and *Paeoniae Radix Alba*. Subsequently, after researchers confirmed the findings by ELISA assays, their research showed that the herb pair can modulate arachidonic acid metabolism, which helps to alleviate depression.

In toxicology and biomarker studies of TCM, transcriptomics is often integrated with metabolomics for the identification of key pathways and early biomarkers of toxicity in TCM. In addition, the emergence and development of Artificial Intelligence and multi-omics sequencing technologies have provided new tools and support for the research of TCM synergism to advance precision.

## Discussion

With the development of physicochemistry, pharmacokinetics, and pharmacodynamics research, it is increasingly confirmed that the active components and molecular mechanisms of the synergistic effects in TCM compatibility are complex. While certain progress has been made in this field through pharmacodynamic experiments through animal or cell models, transcriptomics, metabolomics, and network pharmacology, there are still limitations.

First, the chemical components of TCM are highly complex and can undergo transformation and metabolism in the body after compatibility. Analyzing individual or a few active components alone cannot fully reflect the overall changes after compatibility. Therefore, a complete fingerprinting system for TCM compound formulas is needed to comprehensively present these components.

Moreover, in the study of the pharmacokinetic processes of TCM, current research often fails to adequately reveal the specific mechanisms by which pharmacokinetic changes affect pharmacodynamics. What presents as pharmacodynamic synergy may in fact reflect an “exposure-amplification effect”: one constituent inhibits the metabolism or transport of another, leading to a several fold increase in the pharmacokinetic parameters of the latter. While this phenomenon can enhance therapeutic efficacy, it concurrently narrows the therapeutic window and elevates the risk of dose-dependent toxicity. Therefore, any observed synergy is best interpreted in the context of both the magnitude of exposure amplification and its associated safety profile. Hence, future pharmacokinetic studies should focus more on the dynamic processes of pharmacodynamics as well as medication toxicity, so as to gain a deeper understanding of the in vivo behavior of TCM components and their association with therapeutic effects.

Next, when studying synergy in pharmacodynamics, most research focuses on the cumulative effects of various components in TCM on multiple proteins or pathways, without specifically exploring the synergistic mechanisms before and after compatibility. Future research can be designed from this perspective. Furthermore, in clinical practice, the ratio of TCM compatibility is a crucial factor affecting efficacy. Some studies have begun to test the synergy of these ratios in terms of pharmacological effects. More research is needed to fill this gap, and future studies should focus on analyzing the changes in physicochemistry and pharmacokinetics at different compatibility ratios.

Notably, while existing research on TCM synergy predominantly focuses on efficacy enhancement, toxicity attenuation is equally indispensable for ensuring the clinical safety and rational application of such combinations. Subsequent studies can address this research gap.

Finally, at the research methodology level, controversies regarding the reproducibility of supramolecular assembly methodologies remain unresolved: different laboratories lack standardized assembly protocols and batch-to-batch quality control for the particle size, ζ-potential, and spectroscopic characteristics of herb pairs. Additionally, existing models (e.g., Caco-2, MDCK-MDR1, TC7) mostly remain confined to the in vitro/animal level, with no extension to clinical dose-exposure–response chain verification—resulting in a scarcity of translational evidence.

It is vital to comprehend the changes in pharmacokinetic and pharmacological pathways under varying conditions described above. Such understanding will elucidate the synergistic effects and compatibility mechanisms inherent in TCM, offering valuable insights for the optimization of TCM compound formulas’ clinical application.

## Data Availability

Data availability is not applicable to this article as no new data were created or analyzed in this study.

## Abbreviations

AGE-RAGE: Advanced glycation end-products and their receptor
AQP2: Aquaporin 2
AKT: Protein kinase B
AMPK: AMP-activated protein kinase
ADME: Absorption, distribution, metabolism, and excretion
AVP: Arginine vasopressin
Cat: Catalpol
CD14: Cluster of differentiation 14
CD62E: Endothelial selectin
CNKI: China national knowledge infrastructure
CYP3A: Cytochrome P450 3A
CYP7B1: Cytochrome P450 7B1
Cur: Curcumin
DN: Diabetic nephropathy
ERK: Extracellular signal-regulated kinase
ERK1/2: Extracellular signal-regulated kinase 1/2
EVO: Evodiine
FTIR: Fourier transform infrared spectroscopy
GERD: Gastroesophageal reflux disease
HBF: Huashi Baidu formula
HIF: Hypoxia-inducible factor
HPLC: High-performance liquid chromatography
JNK: C-Jun N-terminal kinase
LDLR: Low-density lipoprotein receptor
LPS: Lipopolysaccharide
Log: Loganin
MAPK: Mitogen-activated protein kinase
mTOR: Mammalian target of rapamycin
MRPs: Multidrug resistance-associated proteins
MRP2: Multidrug resistance protein 2
NET: Neutrophil extracellular trap
P2RY12: Purinergic receptor P2Y12
P-gp: P-Glycoprotein
PCSK9: Proprotein convertase subtilisin/kexin type 9
PPARγ: Peroxisome proliferator-activated receptor gamma
Res: Resveratrol
SphK1: Sphingosine kinase 1
STAT3: Signal transducer and activator of transcription 3
TAS2Rs: Taste receptor type 2
TCM: Traditional Chinese medicine
TRPV1: Transient receptor potential vanilloid 1
UC: Ulcerative colitis
VCMN: Vascular cell-derived microparticles
YF: Yinxieling formula
